# Altering Mucus Rheology to “Solidify” Human Mucus at the Nanoscale

**DOI:** 10.1371/journal.pone.0004294

**Published:** 2009-01-28

**Authors:** Samuel K. Lai, Ying-Ying Wang, Richard Cone, Denis Wirtz, Justin Hanes

**Affiliations:** 1 Department of Chemical & Biomolecular Engineering, The Johns Hopkins University, Baltimore, Maryland, United States of America; 2 Department of Biomedical Engineering, The Sidney Kimmel Comprehensive Cancer Center, Johns Hopkins University School of Medicine, Baltimore, Maryland, United States of America; 3 Department of Biophysics, The Johns Hopkins University, Baltimore, Maryland, United States of America; 4 Department of Institute for NanoBioTechnology, The Johns Hopkins University, Baltimore, Maryland, United States of America; 5 Department of Oncology, The Sidney Kimmel Comprehensive Cancer Center, Johns Hopkins University School of Medicine, Baltimore, Maryland, United States of America; Dalhousie University, Canada

## Abstract

The ability of mucus to function as a protective barrier at mucosal surfaces rests on its viscous and elastic properties, which are not well understood at length scales relevant to pathogens and ultrafine environmental particles. Here we report that fresh, undiluted human cervicovaginal mucus (CVM) transitions from an impermeable elastic barrier to non-adhesive objects sized 1 µm and larger to a highly permeable viscoelastic liquid to non-adhesive objects smaller than 500 nm in diameter. Addition of a nonionic detergent, present in vaginal gels, lubricants and condoms, caused CVM to behave as an impermeable elastic barrier to 200 and 500 nm particles, suggesting that the dissociation of hydrophobically-bundled mucin fibers created a finer elastic mucin mesh. Surprisingly, the macroscopic viscoelasticity, which is critical to proper mucus function, was unchanged. These findings provide important insight into the nanoscale structural and barrier properties of mucus, and how the penetration of foreign particles across mucus might be inhibited.

## Introduction

Mucus is a highly viscous and elastic barrier that protects mucosal surfaces by selectively trapping and shedding pathogens, toxins, and ultrafine particles [1]–[3], while allowing rapid flux of nutrients, antibodies, and cells of the mucosal immune system [4]–[6]. Under shear, mucus becomes a low viscosity lubricant that prevents intra-organ adhesion and enables fundamental processes, including peristalsis, blinking, and copulation [3]. The selective permeability and dynamic viscoelastic behavior of mucus are biochemically regulated by mucins in concert with lipids, salts, cells, DNA, and other macromolecules [7], [8], and strongly varies across length scales. However, classical rheological techniques are incapable of probing viscous and elastic characteristics at submicron length scales.

The mechanical properties of biopolymer networks are accessible by optical-based microrheology techniques [9]–[14]. The time scale-dependent displacements of non-interacting beads allow quantitative measurements of the frequency-dependent local viscous (G″(ω)) and elastic (G′(ω)) forces impeding their Brownian motions [9], [10], [15], [16]. Here, we used fresh, undiluted and unmanipulated human cervicovaginal mucus (CVM), obtained from female donors with healthy vagina flora, as a model of physiological mucus ex vivo [17]. Using high resolution multiple-particle tracking [9], [18]–[20], we observed the translational movements of hundreds of fluorescent probe beads coated with a non-mucoadhesive surface [21], [22], ranging from 100 to 1,000 nm in diameter, and determined the viscoelastic barrier properties of CVM at length scales relevant to pathogens.

## Results and Discussion

**Figure 1:**
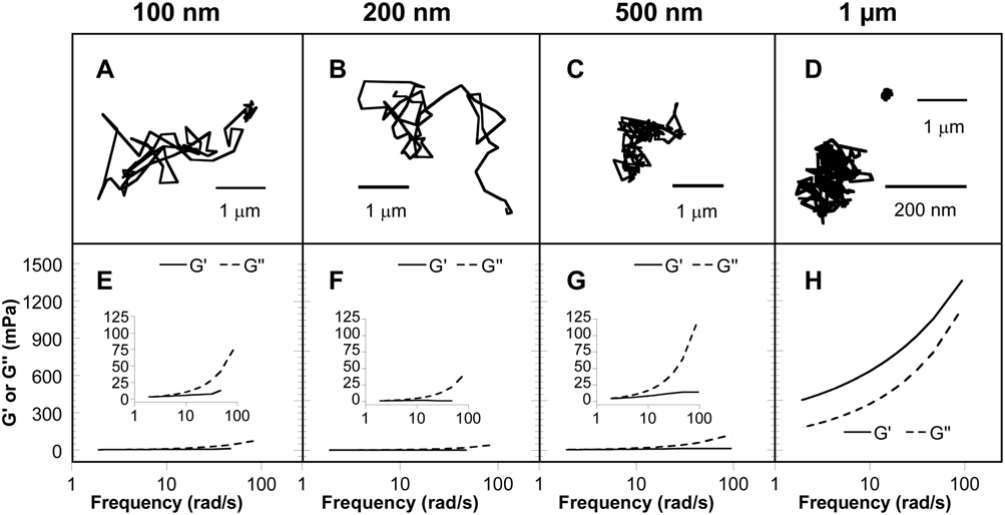
Nanoscale viscoelastic properties of fresh, undiluted human cervicovaginal mucus (CVM). (*A–D*) Representative trajectories of 100 nm (*A*), 200 nm (*B*), 500 nm (*C*), and 1,000 nm (*D*) probe particles in CVM traced over 20 s. Particles exhibit an effective diffusivity within one s.e.m. of the ensemble mean. (*E–H*) Local elastic (G′, solid lines) and viscous (G″, dashed lines) moduli as a function of frequency for the same probe particles: 100 nm (*E*), 200 nm (*F*), 500 nm (*G*), and 1,000 nm (*H*).

We first studied non-mucoadhesive beads 100 to 500 nm in diameter, which represent the relevant sizes for most viruses, environmental ultrafine particles, and synthetic drug delivery systems [21], [23], and compared their motions to those of non-mucoadhesive beads 1 µm in diameter, which are similar in size to bacteria and toxins, such as anthrax. The Brownian trajectories for 100–500 nm beads, probing distances far larger than their sizes over 20 s movies, suggest that mucus acts as a low viscosity fluid at length scales up to 500 nm (Fig. 1*A–C* and Videos S1, S2, S3). In contrast, the constrained nature of the time lapse traces of 1 µm beads is indicative of the elevated mechanical response of a viscoelastic solid that impedes particle motion (Fig. 1*D* and Video S4). Indeed, to beads 500 nm and smaller, CVM poses a low elastic modulus (G′) at all frequencies probed, with an average G′ as low as 1.0 and 4.3 mPa on 200 and 500 nm beads at the lowest shear frequency measured, respectively (Fig. 1*E–G*). In comparison, the effective G′ exerted by CVM on 1 µm beads and the bulk G′ of CVM are 400 and 154,000 mPa at the same shear frequency, respectively (Fig. 1*H*). To submicron entities, CVM exhibits a distinctly higher viscous modulus than elastic modulus (Fig. 1*E–G*), another indication that it behaves as a viscoelastic liquid for 100–500 nm particles. The phase angle, δ, of CVM, defined as tan δ = G″/G′, becomes larger with increasing frequency, reflecting a progressively more liquid-like behavior as expected for a shear-thinning polymer network. In contrast, the elastic modulus of CVM probed by 1 µm beads is significantly higher than the viscous modulus (Fig. 1*H*). These results suggest that unsheared CVM transitions from an impermeable viscoelastic solid at length scales 1 µm or larger to a highly permeable viscoelastic liquid at length scales 500 nm and below.

A simple lattice model of individual mucin fibers in a mucus mesh, based on the biochemistry of mucin fibers and mucin content [3], yields an estimate of ∼60 nm for the average mesh spacing between fibers (Supporting Information Text S1). Since 200 and 500 nm beads diffuse in CVM largely unhindered, the structural elements of physiological CVM are expected to include largely bundled or aggregated mucin fibers, with many spacings ≥500 nm. The rheology of mucus is governed by both viscous drag from interstitial fluids within the mesh spacings and elastic recoil from the mesh fibers. Thus, the low viscosity behavior of CVM at submicron length scales can likely be attributed to low elastic contributions by the mucus mesh. At short time scales (high frequencies), beads sufficiently small relative to the mesh spacing will only experience the viscous drag of the low viscosity interstitial fluid. At longer time scales, steric obstruction by the CVM mesh contributes to an increasing effective viscosity and elastic behavior, but remains within the realm of a permeable viscoelastic liquid. The highly constrained movements of 1 µm beads are likely the consequence of strong steric hindrance and substantial elastic recoil by the CVM mesh, suggesting that the average mesh spacings must be below 1 µm. At larger length scales (up to the bulk rheology), the viscoelastic behavior of CVM increasingly reflects the mechanical strength of the mucus fibers.

**Figure 2:**
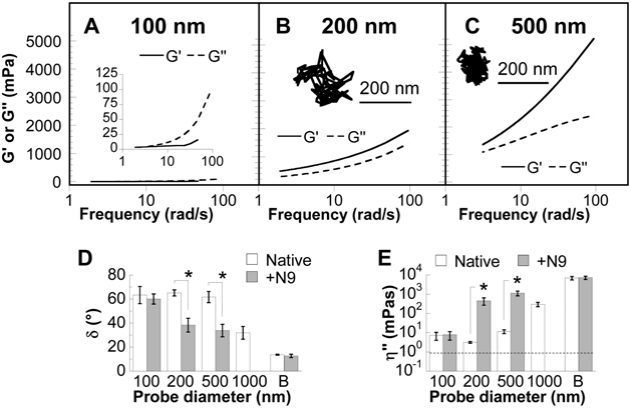
Effect of nonoxynol-9 (N9) on the nanoscale viscoelastic properties of fresh human cervicovaginal mucus (CVM). (*A–C*) Local elastic (G′) and viscous (G″) moduli as a function of frequency for 100 nm (*A*), 200 nm (*B*), and 500 nm (*C*) probe particles in N9-treated CVM. Representative trajectories of 200 nm (*B* inset) and 500 nm (*C* inset) probe particles, with an effective diffusivity within one s.e.m. of the ensemble average. (*D*) Phase angle (δ) at a frequency of 2π rad/s for probe particles in native or N9-treated CVM compared to bulk values (“B”) at the same frequency (mean±s.e.m.). The phase angle for a purely viscous fluid is 90°, while that for a purely elastic solid is 0°. (*E*) Dynamic viscosity (η″) at a frequency of 2π rad/s for probe particles in native or N9-treated CVM compared to bulk values (“B”) at the same frequency (mean±s.e.m.). The dashed line represents the viscosity of water. * denotes statistical significance (P<0.05).

The bundling and aggregation of mucin fibers may be driven by hydrophobic interactions between naked protein or lipid-coated domains along the fibers [3], [21], [24]. Therefore, we hypothesized that the nonionic detergent nonoxynol-9 (N9), a spermicide and microbicide widely used in vaginal gels, lubricants, and condoms [25], may alter the structure and rheology of CVM. We treated the same CVM samples with N9 at minimal dilution but physiological concentration (∼1% by volume dilution of CVM with 10% N9 to a final N9 concentration of 0.1%), and measured the local elastic and viscous moduli of N9-treated CVM at submicron length scales. N9 treatment of CVM led to greatly hindered motions for 200 and 500 nm beads and increased the viscoelastic moduli they probed, but did not significantly affect 100 nm beads (Fig. 2*A–C* and Videos S5, S6, S7). Addition of the same volume of saline instead of N9 did not affect the motions of 200 and 500 nm particles in mucus (visual observations; data not shown). At the 200–500 nm length scales, CVM shifts from behaving as a viscoelastic liquid (phase angle in excess of 60°) to a viscoelastic solid (phase angle ∼35°) upon N9 treatment (Fig. 2*D*). The liquid-solid transition is also reflected by a ∼100–150-fold increase in the dynamic viscosity, η″ = G″/ω, experienced by 200 and 500 nm beads (Fig. 2*E*). The rapid transport of 100 nm beads under either condition (with or without N9 treatment) indicates that N9 did not cause beads to adhere to CVM. Thus, the induced changes in the rheology at selected length scales are likely due to a marked reduction in the mesh spacing, subjecting the 200 and 500 nm beads to viscoelastic steric constraints by the structural elements of CVM. Since N9-induced changes in mesh microstructure did not perturb the movements of 100 nm beads, N9-treated CVM remains a low viscosity fluid at a length scale of 100 nm. These results highlight the ability to chemically transform the size-dependent barrier properties of CVM by greatly reducing the critical length scale for its viscoelastic solid-liquid transition.

**Figure 3:**
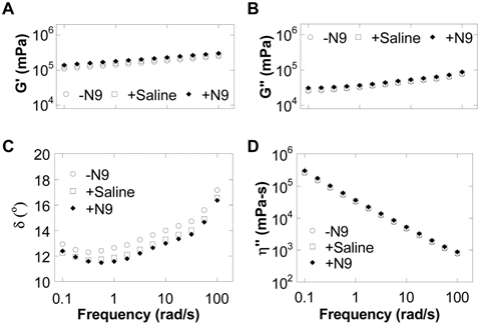
Macrorheological characterization of fresh human cervicovaginal mucus (CVM) under dynamic oscillatory shear. (*A–D*) Elastic modulus (G′) (*A*), viscous modulus (G″) (*B*), phase angle (δ) (*C*), and dynamic viscosity (η″) (*D*) for untreated (−N9), saline-treated (+Saline), or nonoxynol-9-treated (+N9) CVM.

Since N9 markedly altered the nanoscale rheology of CVM, we next tested if the bulk rheology was likewise perturbed. The bulk rheology of human mucus (i.e., its response to shear) is critical to maintaining proper mucus clearance rates in the body and the ability of mucus to act as a lubricant [3], [26]–[29]. The bulk viscous and elastic moduli were quantified by measuring the torque required to apply a small, fixed-amplitude oscillatory stress at specified frequencies using a sensitive strain-controlled cone-and-plate rheometer [23], [30]. Surprisingly, the bulk viscous and elastic moduli of mucus were both minimally changed upon N9 treatment (Fig. 3*A,B*). Macroscopically, native and N9-treated CVM both behaved as a highly viscoelastic gel, with a low phase angle even under relatively high shear (Fig. 3*C*). CVM in both conditions also exhibited log-linear shear-thinning of viscosity, a classical feature of mucus secretions [3], [21], [23], [31]. Thus, N9 altered only nanoscale mechanical properties of mucus without significantly affecting the bulk, macroscale rheological properties.

**Figure 4:**
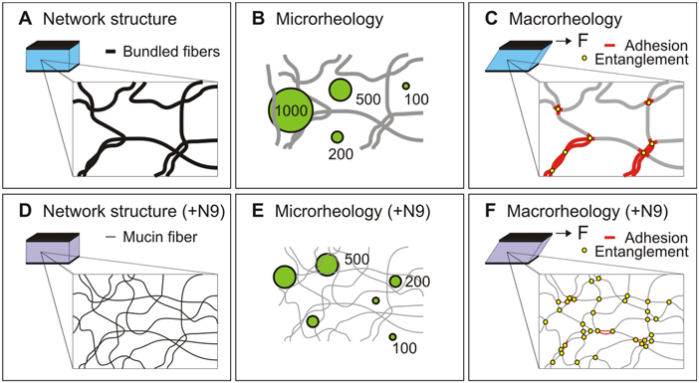
Summary of the interpretation of results. (*A*) The mesh structure of native human cervicovaginal mucus (CVM) consists of individual mucin fibers bundled together, leading to large mesh spacings. (*B*) The microrheology of CVM, quantified using different sized probe nanoparticles, suggests that CVM is largely a viscoelastic fluid at length scales 500 nm or below, whereas at length scales 1,000 nm or higher the mesh elements contribute to a markedly greater local elasticity characteristic of viscoelastic solids. (*C*) The macrorheology of CVM reflects contributions from entanglements as well as hydrophobic adhesive interactions between the mesh elements. (*D*) Treatment of CVM with nonoxynol-9 (N9) leads to unbundling of the mesh elements and significantly reduced mesh spacings, due to reduced hydrophobic interactions between mucin fibers. (*E*) The microrheology of N9-treated CVM becomes that of a viscoelastic solid at length scales down to 200 nm, but remains largely unperturbed at length scales ∼100 nm or below. (*F*) The effect of N9 cannot be probed by macrorheology, as the reduction in adhesive interactions by N9 is likely balanced by increased entanglements between mucin fibers.

In classical theories of polymer network mechanics [32]–[34], the bulk elastic modulus of concentrated isotropic solutions of entangled semiflexible polymers is described by G′∼(κ^2^ ξ^−2^ L_e_^−3^)/kT, where ξ represents the mesh size, L_e_ the chain segment length, k_B_T the thermal energy, and κ is related to the persistence length of the chain, L_p_, by L_p_≈κ/k_B_T. Models for the elastic behavior of stiff and crosslinked networks [33] predict G′∼(κ ^2^ ξ^−5^)/k_B_T, and for networks of flexible chains G′∼k_B_T ξ^−3^. Assuming only ξ changes, a hypothetical 3-fold reduction in the average mesh spacing of CVM would be expected to increase the measured bulk elasticity, G′, by ∼9, ∼250, or ∼27–fold depending on whether CVM is modeled as a semiflexible, stiff and crosslinked, or flexible polymer network, respectively. Since the bulk elasticity was only minimally perturbed, classical models of polymer networks fail to describe the molecular interactions contributing to the bulk mechanical properties of CVM. To understand this, consider that the bulk rheology of CVM likely reflects a combination of entropic contributions arising from the entanglement of mucin mesh elements [35], and enthalpic contributions from adhesive interactions between the lipid-coated hydrophobic domains of mucins [36], [37] and disulphide crosslinks. Enthalpic contributions are not considered in classical theories (which would predict that N9 would strongly increase the bulk viscoelasticity due to greater entropic contributions from the increased entanglements of unbundled mucin fibers). However, the detergent nature of N9 likely compensates for the expected entanglement-driven increase in the mechanical properties of CVM by strongly reducing adhesive hydrophobic interactions between mucin fibers and bundles. These two opposing effects offset each other to the extent that even a sensitive cone and plate rheometer is unable to detect the structural and nanoscale rheological changes to mucus caused by N9 (Fig. 4). We do not expect that N9-treatment of CVM eliminated adhesive interactions between mucins at the N9 concentrations used here. This can be inferred by the unperturbed viscoelasticity of CVM as probed by 100 nm beads in the presence of N9, which suggests that the effective mesh spacing is still markedly larger than the estimated mesh spacing from a lattice model of individual, unbundled mucin fibers. Thus, mucin fibers likely remain partially bundled in N9-treated CVM in this study.

The viscoelastic behavior of mucus, and perhaps other biopolymer networks, changes sharply based on the length scale of interest. Importantly, we demonstrate here that the nanoscale rheology of mucus may be altered, without affecting the macrorheological properties that are essential to the proper functioning of mucus as a lubricant and proper mucus clearance. Although it is speculative, selectively altering the nanoscale rheology of mucus without changing its bulk rheology warrants investigation as a potential prophylactic strategy to prevent infections by reducing the mucus-penetration of pathogens.

## Materials and Methods

### Human cervicovaginal mucus (CVM) collection

CVM was collected as previously described [17], [21]. Briefly, undiluted cervicovaginal secretions from women with normal vaginal flora were obtained using a self-sampling menstrual collection device following a protocol approved by the Institutional Review Board of the Johns Hopkins University. Written informed consent was obtained from all participants. The device was inserted into the vagina for ∼30 s, removed, and placed into a 50 mL centrifuge tube. Samples were centrifuged at 1,000 rpm for 2 min to collect the secretions. Mucus samples were visually observed for a substantial increase in vaginal secretion volume and a marked reduction in the viscoelasticity, hallmarks of mucus from women with abnormal vaginal flora (e.g. women with bacterial vaginosis) [38].

### Macrorheological characterization

To allow comparison with our microrheological characterization, macrorheological characterization of CVM was performed with a strain-controlled cone and plate rheometer (ARES-100, Rheometrics, Piscataway, NJ) using techniques described previously [23]. Cervicovaginal mucus from 4–5 different donors was pooled (total volume ∼1.3 mL), stored at 4°C and used within 8 hr of collection. The temperature of specimens was maintained at 37°C during measurements. Oscillatory deformations of small amplitude (1% strain) and controlled frequency were applied to extract the frequency-dependent viscoelastic properties with minimal shearing damage to the CVM. We report the frequency-dependent elastic and viscous moduli, G′(ω) and G″(ω), which are the in-phase and out-of-phase components, respectively, of the stress induced in CVM samples divided by the maximum amplitude of the applied deformation. The rheology of untreated, saline-treated, and N9-treated CVM was evaluated in sequential order, with saline or 10% N9 added at 1% volume of the sample, gently stirred, and incubated for 17 min prior to subsequent measurements. Three independent experiments were performed.

### Preparation and characterization of non-mucoadhesive microrheology beads

Yellow-green (Ex/Em 505/515 nm) or red (Ex/Em 580/605 nm) fluorescent carboxyl-modified polystyrene particles sized 100, 200, 500, and 1,000 nm (Molecular Probes, Eugene, OR) were covalently modified with amine-terminated polyethylene glycol (molecular mass, 2–3.4 kDa; Nektar Therapeutics, San Carlos, CA) by a carboxyl-amine reaction in 6∶1 excess, as previously described [21]. Size and ζ-potential were determined by dynamic light scattering and laser Doppler anemometry, respectively, using a Nanosizer ZS90 (Malvern Instruments, Southborough, MA). Size measurements were performed at 25°C at a scattering angle of 90°, and measurements were performed according to instrument instructions. For 1,000 nm beads, the size was determined by transmission electron microscopy using a Philips 420 electron microscope (N. V. Phillips). Size and surface charge measurements are available in Table S1. The neutral surface charge of all particles is in good agreement with a fluorimetric assay [22] that estimated the number of PEG molecules on the surface of the particles to be in excess of 1 molecule of PEG per nm^2^.

### Multiple particle tracking

Particle transport rates were measured by analyzing trajectories of yellow-green or red fluorescent particles, recorded using a silicon-intensified target camera (VE-1000, Dage-MTI, Michigan, IN) mounted on an inverted epifluorescence microscope (Zeiss, Thornwood, NY) equipped with a 100× oil-immersion objective (N.A., 1.3) and the appropriate filters. Experiments were carried out in 8-well glass chambers (LabTek, Campbell, CA), where diluted particle solutions (0.0082% wt/vol) were added to 250–500 µL of fresh mucus to a final concentration of 3% v/v (final particle concentration, 8.25×10 ^−7^ wt/vol) and incubated at 37°C for 2 hr before microscopy. After microscopy of beads in untreated CVM, 10% N9 was added to mucus at 1% volume, gently stirred, and again incubated at 37°C for 2 hr prior to microscopy. Trajectories of n≥120 particles were analyzed for each experiment, and three independent experiments were performed for each condition. Movies were captured with MetaMorph software (Universal Imaging, Downingtown, PA) at a temporal resolution of 66.7 ms for 20 s. The tracking resolution was 10 nm, as determined by tracking the displacements of particles immobilized with a strong adhesive [9]. The image size was 512×512 pixels with ∼0.23 µm/pixel and a 16-bit image depth. The amplifier gain was adjusted to avoid signal saturation. The coordinates of nanoparticle centroids were transformed into time-averaged mean-squared displacement (MSD), calculated as <Δr^2^(τ)> = <[x(t+τ)−x(t)]^2^+[y(t+τ)−y(t)]^2^>, where x(t) and y(t) represent the nanoparticle coordinates at a given time and τ is the time scale or time lag [20], [39]. Distributions of MSDs and effective diffusivities were calculated from this data, as demonstrated previously [21], [22].

### Microrheological characterization

Microrheological data was extracted from the amplitude and time scale-dependence of the geometrically averaged ensemble mean square displacements of the particles [9], [15], [16], [23]. These values can be used to calculate the viscoelastic spectrum G(s) = 2k_B_T/3πas<Δr^2^(s)>, where k_B_ is the Boltzmann constant, T is the absolute temperature, and a is the particle radius [10], [34]. Here, s represents the Laplace frequency, and <Δr^2^(s)> is the unilateral Laplace transform of <Δr^2^(τ)>. The Fourier transform equivalent of G(s) is the complex shear modulus G*(ω), from which the elastic modulus G′(ω) and viscous modulus G″(ω) can be calculated. Thus, time scale-dependent MSD can be directly related to the traditional frequency-dependent elastic and viscous moduli. Readers are referred to existing literature for further details [9], [15], [16], [23]. All microrheological data is reported as a function of shear frequency in units of rad/s for easy comparison with macrorheological values. Since observations of native and N9-treated mucus were performed in the same sample, a one-tailed, paired t-test was used to evaluate statistical significance at an alpha level of 0.05.

## Supporting Information

Video S1 Transport of 100 nm non-mucoadhesive probe beads in native human cervicovaginal mucus over the course of 20 s (shown at 2× speed). The trajectories of the beads are largely unhindered and Brownian.
(0.78 MB MOV)

Video S2 Transport of 200 nm non-mucoadhesive probe beads in native human cervicovaginal mucus over the course of 20 s (shown at 2× speed). The trajectories of most beads are largely unhindered and Brownian.
(0.55 MB MOV)

Video S3 Transport of 500 nm non-mucoadhesive probe beads in native human cervicovaginal mucus over the course of 20 s (shown at 2× speed). The trajectories of most beads are largely unhindered and Brownian.
(0.27 MB MOV)

Video S4 Transport of 1 µm non-mucoadhesive probe beads in native human cervicovaginal mucus over the course of 20 s (shown at 2× speed). The bead motions are highly constrained and non-Brownian.
(0.10 MB MOV)

Video S5 Transport of 100 nm non-mucoadhesive probe beads in nonoxynol-9-treated human cervicovaginal mucus over the course of 20 s (shown at 2× speed). The trajectories of the beads are largely unhindered and Brownian.
(0.99 MB MOV)

Video S6 Transport of 200 nm non-mucoadhesive probe beads in nonoxynol-9-treated human cervicovaginal mucus over the course of 20 s (shown at 2× speed). The trajectories of most beads are highly constrained and non-Brownian.
(0.21 MB MOV)

Video S7 Transport of 500 nm non-mucoadhesive probe beads in nonoxynol-9-treated human cervicovaginal mucus over the course of 20 s (shown at 2× speed). The trajectories of most beads are highly constrained and non-Brownian.
(0.11 MB MOV)

Text S1 (0.04 MB DOC)

Table S1 (0.03 MB DOC)
