# Supplementary material for: Altering Mucus Rheology to “Solidify” Human Mucus at the Nanoscale
Source: PLoS One. 2009 Jan 28;4(1):e4294. doi: 10.1371/journal.pone.0004294 (PMC2627937; doi:10.1371/journal.pone.0004294)
Supplement: Text S1 — (0.04 MB DOC) [file pone.0004294.s008.doc]

**Supporting Information**

**Altering Mucus Rheology to “Solidify” Human Mucus at the Nanoscale**

Samuel K. Lai^*^, Ying-Ying Wang^*^, Richard Cone, Denis Wirtz, Justin Hanes^†^

^*^ These authors contributed equally to this work.

^†^ To whom correspondence should be addressed. Email: hanes@jhu.edu

**Supporting Text**

**Cubic lattice model of mucus**

**Definitions:**

ξ

b

Bundle

Cross-section

Mucin Fiber Bundles

b

b = bundle diameter

ξ = mesh spacing

v = volume of each bundle

c = fiber volume concentration

d = cube length

l = length of bundle

We model mucus as a simple cubic lattice. The 12 edges of a single cubic unit of the lattice are composed of segments of mucin fiber bundles, with ¼ of each bundle’s volume contained within the cube. The volume of the fiber bundles can therefore be related to the fiber volume concentration and the volume of the cube by 12(v/4) = cd^3^.

Each bundle’s volume is given by v = π(b/2)^2^l, where l = d and d = b + ξ in the case of a single cubic unit. By substituting these equations and rearranging, we obtain the following quadratic:

ξ^2^ + 2bξ + (1-¾π/c) b^2^ = 0.

Solving for the mesh spacing gives the following equation, where mesh spacing must be positive:

.

If we consider single mucin fibers that are not bundled, then b = 7 nm, the approximate diameter of individual mucin fibers based on biochemical analysis [1]. We also estimate a mucin fiber concentration of c = 2.5 % v/v, which is on the lower end of the typical range of mucin concentrations [2] (higher concentrations would lead to an even smaller mesh spacing). These values yield a mesh spacing estimate of ~60 nm.

For comparison, we can also estimate the average mesh spacing by assuming that the mucin mesh is a flexible polymer network. In a good solvent, the mesh size, ξ, of a flexible polymer network is described by:

where R_g_ is the radius of gyration, c is the polymer concentration, and c* is defined by:

where M_w_ is the molecular weight of the polymer and N_a_ is Avogadro’s number [3]. Using published values of M_w_ and R_g_ for human cervical mucus glycoproteins [4], and a mucin fiber concentration of 2.5%, we estimate that the mesh spacing for a flexible mucin network is ~25 nm, which is in good agreement with the above estimate.

**References**

1. Olmsted SS, Padgett JL, Yudin AI, Whaley KJ, Moench TR, et al. (2001) Diffusion of macromolecules and virus-like particles in human cervical mucus. Biophys J 81: 1930-1937.

2. Cone R (1999) Mucus. In: Lamm ME, Strober W, McGhee JR, Mayer L, Mestecky J et al., editors. Mucosal Immunology. 3 ed. San Diego: Academic Press. pp. 43-64.

3. Biehl R, Guo X, Prud'homme RK, Monkenbusch M, Allgeier J, et al. (2004) Diffusion of compact macromolecules through polymer meshes: mesh dynamics and probe dynamics. Phys B 350: 76-78.

4. Sheehan JK, Carlstedt I (1984) Hydrodynamic properties of human cervical-mucus glycoproteins in 6M-guanidinium chloride. Biochem J 217: 93-101.
