# Supplementary material for: Altering Mucus Rheology to “Solidify” Human Mucus at the Nanoscale
Source: PLoS One. 2009 Jan 28;4(1):e4294. doi: 10.1371/journal.pone.0004294 (PMC2627937; doi:10.1371/journal.pone.0004294)
Supplement: Table S1 — (0.03 MB DOC) [file pone.0004294.s009.doc]

**Table S1. Characterization of non-mucoadhesive probe beads.**

| **Size (nm)*** | **Diameter (nm)**^†^ | **ζ-potential (mV)**^†^ |
| --- | --- | --- |
| 100 | 114 ± 4 | -2.5 ± 1.2 |
| 200 | 231 ± 6 | -2.2 ± 2.2 |
| 500 | 515 ± 17 | -3.9 ± 3.1 |
| 1,000 | 1,050^‡^ | -3.9 ± 2.8 |

* Provided by the manufacturer.

^†^ Mean ± S.D.

^‡^ Estimated by transmission electron microscopy (see Materials and Methods for details).
